# Magneto-hydrodynamic peristaltic flow of a Jeffery fluid in the presence of heat transfer through a porous medium in an asymmetric channel

**DOI:** 10.1038/s41598-023-48137-x

**Published:** 2023-11-30

**Authors:** A. M. Abd-Alla, S. M. Abo-Dahab, Doaa. M. Salah, F. S. Bayones, M. A. Abdelhafez

**Affiliations:** 1 Department of Mathematics, Faculty of Science, Sohag University, Sohag, Egypt; 2 Department of Mathematics, Faculty of Science, South Valley University, Qena, 83523 Egypt; 3 Department of Mathematics and Statistics, College of Science, Taif University, P. O. Box 11099, 21944 Taif, Saudi Arabia

**Keywords:** Biophysics, Mathematics and computing

## Abstract

In the present paper, the effects of magnetic field and heat transfer on the peristaltic flow of a Jeffery fluid through a porous medium in an asymmetric channel have been studied. The governing non-linear partial differential equations representing the flow model are transmuted into linear ones by employing the appropriate non-dimensional parameters under the assumption of long wavelength and low Reynolds number. Exact solutions are presented for the stream function, pressure gradient, and temperature. The frictional force and pressure rise are both computed using numerical integration. Using MATLAB R2023a software, a parametric analysis is performed, and the resulting data is represented graphically. For all physical quantities considered, numerical calculations were made and represented graphically. Trapping phenomena are discussed graphically. The obtained results can be applied to enhance pumping systems in engineering and gastrointestinal functions. This analysis permits body fluids such as blood and lymph to easily move inside the arteries and veins, allowing oxygen supply, waste elimination, and other necessary elements.

## Introduction

Peristaltic transport has become a prominent topic for academics in recent years due to its use in physics, applied mathematics, physiology, engineering, various hose pumps, etc. Peristalsis is defined as a series of muscle relaxations and contractions of a blood vessel wall that push materials forward in fluid through tubules in a wave-like motion without an interface with the pump parts. Peristaltic movement can be observed during the passage of food through the stomach, esophagus, and intestines, through the blood in the arteries, veins, and capillaries, the exit of urine through the ureters from the kidney to the bladder, the movement of the egg and embryo in the tubes, and so on. Toxic fluids are transported in nuclear facilities using the peristaltic phenomenon. Such biomedical applications motivate the researchers and, therefore, many theoretical investigations have been tackled to see the impact of magnetic field on peristaltic flows^1–4^. A porous medium is the matter which contains a number of small holes distributed throughout it. The fluid transport through porous medium is widely applicable in the vascular beds, lungs, kidneys, tumorous vessels, bile duct, gall bladder with stones, and small blood vessels. Some scientists contributed to study and develop the peristaltic flow through a porous medium^5–8^. Latham^9^ studied the peristaltic mechanism to investigate urine flow through the ureter. In these days, the endoscope is a very significant tool utilize for analysing causes responsible for various complication in the organs of human in which the fluid is carried by peristaltic pumping like stomach and small intestine. There is no difference between catheter and an endoscope from dynamic point of view. Furthermore, the injection of a catheter will change the distribution and flow field in an artery^10^. Electroosmotic transport is a vital concern in microfluidics because of its applications in healthcare research, scientific and biological analysis, cardiac output challenges, medicine, and the treatment of aberrant and sickle cell diseases^11–14^. Nanoparticle research is a topic of intense scientific interest due to the wide variety of possible applications in the biological, optical, and electrical fields. Nanoparticle examination is in the blink of an eye a region of effective experimental enthusiasm because of a gigantic scope of potential applications in electronic, optical, biomedical field. These tiny particles are mostly found in the metals such as nitrides, Carbides or non-metals (Graphite, Carbon nanotubes)**,** nitrides^15–21^. Ayub and Shahzadi^22^ analyzed a ballon model with Cu-blood medicated nanoparticles as drug agent through overlapped curved stenotic artery having compliant walls. Due to their wide range of industrial and engineering applications, such as the sketch of plastic films, food mixing, nuclear waste storage, aerodynamics, composite materials, paper and milk production, metal spinning processes, petroleum production, and many others, non-Newtonian liquids have seen a significant increase in interest in recent years. This inspired the development of several fluid flow, material analysis, and nanomaterials models^23–28^. The bio-convected phenomena occurs due to the upward average movement of microorganisms that are denser in comparison to base-liquids such as water. The aggregation of microorganisms disrupted the suspension of surface by its increased density. This procedure resulted in microorganism tumbling and bio-convection current generation. The motivation for studying bioconvective phenomena in nanomaterials in view of such novel restrictions is to manage the transport process, which is required in engineering and industrial operations. For more details, see^29–32^. Different pragmatic applications experience the flow through a permeable medium especially in geophysical fluid dynamics. Limestone, Sandstone, gall bladder with stones in tiny blood vessels, beach sand, bile duct and the human lung are the important examples of natural porous media^33–38^. Few researchers have developed reduced graphene oxide–metal oxide composite gas sensors with excellent electrical and gas-sensing capabilities. However, it is still a relatively unexplored territory. Kiranakumar et al.^39^ provides an overview of electrical and gas-sensing properties of reduced graphene oxide–metal oxide nanocomposites with improved sensitivity, selectivity, stability, and other sensing performances. As the importance of heat transfer on peristaltic flow in porous media, many attempts have been made by researchers and scientists to study and develop peristaltic transfer due to its inclusion in many important applications. In addition, engineers use finger and roller pumps that operate on the peristaltic principle. ^40–41^discussed the heat transfer and magnetic field on the peristaltic flow. Many industrial and biological instruments, such as cylinder pumps, finger pumps, heart–lung machines, blood pump machines, and dialysis machines, are designed on the basis of the peristaltic mechanism. Recently, Abd-Alla et al.^42^ investigated the effect of heat and mass transfer on the nanofluid of peristaltic flow in a ciliated tube. In Refs.^43–51^, peristaltic flow with new parameters with or without endoscope has been discussed.

The motivation of this paper is to study the magneto-hydrodynamic peristaltic flow of a Jeffery fluid in the presence of heat transfer through a porous medium in an asymmetric channel. Here we examine the impact of magnetic field and heat transfer on peristaltic flow an asymmetric channel. The aim of this paper is to demonstrate the effect of the porosity of the porous medium, thermal radiation, Darcy number and magnetic field in an asymmetric channel. Significant modelling is conferred with the aid of dimensionless parameters and using approximation of low Reynolds number and long wavelength to obtain linearized system of coupled differential equations which are then solved analytically. Finally, the detailed computational results are compiled and discussed with the physical interpretation of our results. The graphical upshots for the velocity, temperature, concentration, the gradient pressure, pressure rise and the friction force are examined for influential parameters. Results acquired from this examination provide a useful understanding and new visions about the particular nature of the peristalsis. Finally, the numerical result displayed by figures and the physical meaning is explained.

## Formulation of the problem

**Figure 1:**
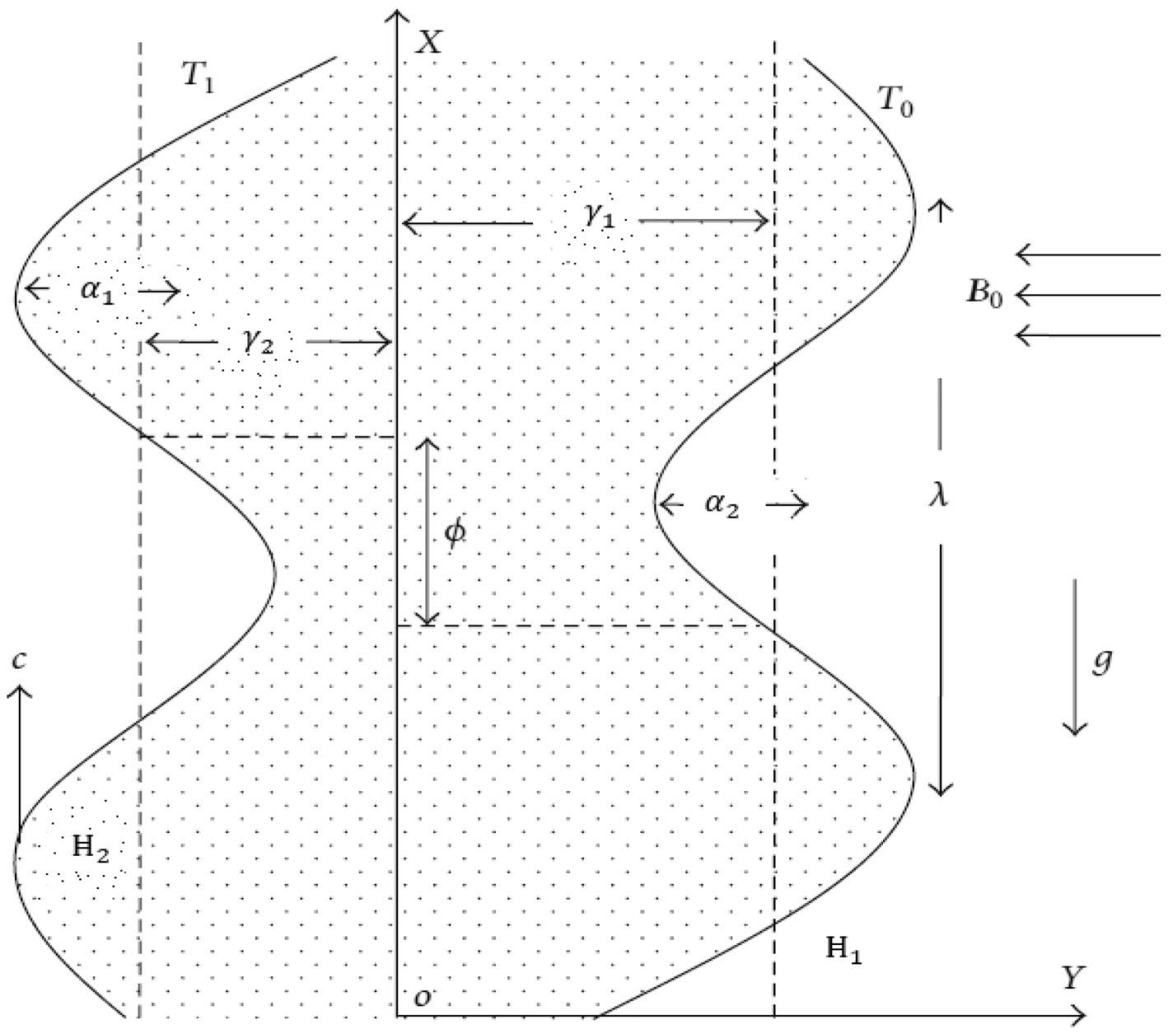
Diagrammatic depiction of the physical model.

Assume we have an asymmetric vertical channel of width ${\alpha }_{1}+{\alpha }_{2}$ in a porous space for an incompressible Jeffrey fluid in the presence of heat when subjected to a magnetic field. The physical model of the problem and the flow coordinate system are shown in Fig. 1. The geometry of peristalsis’s walls is defined as

$${\bar{}\!\!\text{H}}_{1} \left( {X,t} \right) = \alpha_{1} + \gamma_{1} {\text{cos}}\left[ {\frac{2\pi }{\lambda }\left( {X - ct} \right)} \right] , \;{\text{upper wall}},$$

1 $${\bar{}\!\!\text{H}}_{2} \left( {X,t} \right) = - \alpha_{2} - \gamma_{2} {\text{cos}}\left[ {\frac{2\pi }{\lambda }\left( {X - ct} \right) + \phi } \right] , {\text{lower wall}},$$

where the higher and lower waves’ amplitudes, respectively, are ${\gamma }_{1}$, ${\gamma }_{2}$, λ is the wavelength, t time, $\upphi$ is the phase difference belongs to [0, π]. Furthermore, ${\alpha }_{1}$, ${\alpha }_{2}$, ${\gamma }_{1}$, ${\gamma }_{2}$ and $\upphi$ satisfy the inequality below:

2 $${\gamma }_{1}^{2}+{\gamma }_{2}^{2}+2{\gamma }_{1}{\gamma }_{2}{\text{cos}}\upphi \le {\left({\alpha }_{1}+{\alpha }_{2}\right)}^{2} .$$

The governing equations in the laboratory frame are^7,8^

$$\frac{\partial U}{\partial X}+\frac{\partial V}{\partial Y}=0,$$

$$\rho \left(U\frac{\partial U}{\partial X}+V\frac{\partial V}{\partial Y}\right)=-\frac{\partial P}{\partial X}+\frac{1}{\epsilon }\frac{\partial {\tau }_{xx}}{\partial X}+\frac{1}{\epsilon }\frac{\partial {\tau }_{xy}}{\partial Y}-\frac{{\mu }_{^\circ }}{k}U+\rho g{\alpha }_{T}\left(T-{T}_{^\circ }\right)-\sigma {B}_{^\circ }^{2}U,$$

$$\rho \left(U\frac{\partial U}{\partial X}+V\frac{\partial V}{\partial Y}\right)=-\frac{\partial P}{\partial Y}+\frac{1}{\epsilon }\frac{\partial {\tau }_{yx}}{\partial X}+\frac{1}{\epsilon }\frac{\partial {\tau }_{yy}}{\partial Y}-\frac{{\mu }_{^\circ }}{k}V,$$

$$\rho {c}_{p}\left(U\frac{\partial }{\partial X}+V\frac{\partial }{\partial Y}\right)T=K\left(\frac{{\partial }^{2}}{{\partial X}^{2}}+\frac{{\partial }^{2}}{{\partial Y}^{2}}\right)T+{Q}_{^\circ }-\frac{\partial q}{\partial y} .$$

The relationships between the two frames are defined as follows:

3 $$x=X-ct,Y=y,u=U-c,v=V,p\left(x\right)=P\left(X,t\right),$$

where $(u, v), (U, V), (p, P)$ and $T$ represent the components of velocity in the wave frame of reference, the velocity components in laboratory frame, the pressure in wave and fixed frame and, the temperature respectively. Since a small Reynolds number is assumed, the induced magnetic field is ignored.

Following are the basic equations used to explain the flow field:

4 $$\frac{\partial \mathrm{u}}{\partial \mathrm{x}}+\frac{\partial \mathrm{v}}{\partial \mathrm{y}}=0,$$

5 $$\rho \left(\left(u+c\right)\frac{\partial u}{\partial x}+v\frac{\partial v}{\partial y}\right)=-\frac{\partial p}{\partial x}+\frac{1}{\epsilon }\frac{\partial {\tau }_{xx}}{\partial x}+\frac{1}{\epsilon }\frac{\partial {\tau }_{xy}}{\partial y}-\frac{{\mu }_{^\circ }}{k}\left(u+c\right)+\rho g{\alpha }_{T}\left(T-{T}_{^\circ }\right)-\sigma {B}_{^\circ }^{2}\left(u+c\right),$$

6 $$\rho \left(u\frac{\partial u}{\partial x}+v\frac{\partial v}{\partial y}\right)=-\frac{\partial p}{\partial y}+\frac{1}{\epsilon }\frac{\partial {\tau }_{yx}}{\partial x}+\frac{1}{\epsilon }\frac{\partial {\tau }_{yy}}{\partial y}-\frac{{\mu }_{^\circ }}{k}v,$$

7 $$\rho {c}_{p}\left((u+c)\frac{\partial }{\partial x}+v\frac{\partial }{\partial y}\right)T=\left(\frac{{\partial }^{2}}{{\partial x}^{2}}+\frac{{\partial }^{2}}{{\partial y}^{2}}\right)T+{Q}_{^\circ }-\frac{\partial q}{\partial y},$$

where

$${\tau }_{xx}=\frac{2{\mu }_{^\circ }}{1+{\lambda }_{1}}\left[1+{\lambda }_{2}\left(\left(u+c\right)\frac{\partial }{\partial x}+v\frac{\partial }{\partial y}\right)\right]\frac{\partial u}{\partial x},$$

$${\tau }_{xy}=\frac{2{\mu }_{^\circ }}{1+{\lambda }_{1}}\left[1+{\lambda }_{2}\left(\left(u+c\right)\frac{\partial }{\partial x}+v\frac{\partial }{\partial y}\right)\right]\left(\frac{\partial u}{\partial y}+\frac{\partial v}{\partial x}\right),$$

8 $${\tau }_{yy}=\frac{2{\mu }_{^\circ }}{1+{\lambda }_{1}}\left[1+{\lambda }_{2}\left(\left(u+c\right)\frac{\partial }{\partial x}+v\frac{\partial }{\partial y}\right)\right]\frac{\partial v}{\partial y}.$$

In the above equations k is the permeability of the porous medium, ${Q}_{^\circ }$ is the heat source, ${\lambda }_{1}$ is the ratio of relaxation to retardation times, ${\lambda }_{2}$ is the retardation time, ρ is the density, $K_{1}$ is the thermal conductivity, σ is the electrical conductivity of the fluid, ${c}_{p}$ is the specific heat, q is the radiative heat flux and $\epsilon$ is the porosity of the porous medium and ${B}_{^\circ }$ is the applied magnetic field. The non-dimensional quantities are given below:

$$x=\frac{\overline{x}}{\lambda },y=\frac{\overline{y}}{{\alpha }_{1}},u=\frac{\overline{u}}{c },v=\frac{\overline{v}}{c\delta },{h}_{1}=\frac{{\bar{}\!\!\text{H}}_{1}}{{\alpha }_{1}},{h}_{2}=\frac{{\bar{}\!\!\text{H}}_{2}}{{\alpha }_{1}},\tau =\frac{{a}_{1}\overline{\tau }}{{\mu }_{^\circ }c},t=\frac{c\overline{t}}{\lambda },Da=\frac{k}{{\alpha }_{1}^{2}},\delta =\frac{{\alpha }_{1}}{\lambda },$$

$$p=\frac{{\alpha }_{1}^{2}\overline{p}}{{\mu }_{^\circ \lambda }c}, a=\frac{{\gamma }_{1}}{{\alpha }_{1}},b=\frac{{\gamma }_{2}}{{\alpha }_{1}},d=\frac{{\alpha }_{2}}{{\alpha }_{1}},Gr=\frac{\rho g{\alpha }_{T}{\alpha }_{1}^{2}\left({T}_{1}-{T}_{^\circ }\right)}{{\mu }_{^\circ }c},Re=\frac{\rho c{\alpha }_{1}}{{\mu }_{^\circ }},\theta =\frac{\overline{T }-\overline{{T }_{^\circ }}}{{T}_{1}-\overline{{T }_{^\circ }}},$$

9 $$\beta =\frac{{Q}_{^\circ }{\alpha }_{1}^{2}}{k\left({T}_{1}-{T}_{^\circ }\right)},M=\sqrt{\frac{\sigma }{{\mu }_{^\circ }}}{B}_{^\circ }{\alpha }_{1}, q=-R{K}_{1}\frac{\partial T}{\partial y}.$$

Applying (9) in (1) and (4)–(8) and illuminate the bars, we have,

10 $$\frac{\partial \mathrm{u}}{\partial \mathrm{x}}+\frac{\partial \mathrm{v}}{\partial \mathrm{y}}=0,$$

11 $$Re\delta \left(\left(u+1\right)\frac{\partial u}{\partial x}+v\frac{\partial v}{\partial y}\right)=-\frac{\partial p}{\partial x}+\frac{\delta }{\epsilon }\frac{\partial {\tau }_{xx}}{\partial x}+\frac{1}{\epsilon }\frac{\partial {\tau }_{xy}}{\partial y} -\frac{1}{Da}\left(u+1\right)+Gr\theta -{M}^{2}\left(u+1\right),$$

12 $$Re{\delta }^{3}\left(\left(u+1\right)\frac{\partial v}{\partial x}+v\frac{\partial v}{\partial y}\right)=-\frac{\partial p}{\partial y}+\frac{{\delta }^{2}}{\epsilon }\frac{\partial {\tau }_{xy}}{\partial x}+\frac{\delta }{\epsilon }\frac{\partial {\tau }_{yy}}{\partial y}-\frac{{\delta }^{2}}{Da}v,$$

13 $$\rho {c}_{p}\frac{c\delta }{{a}_{1}}\left(\left(u+1\right)\frac{\partial }{\partial x}+v\frac{\partial }{\partial y}\right)\theta \left({T}_{1}-{T}_{^\circ }\right)=\left(\frac{{\delta }^{2}}{{a}_{1}^{2}}\frac{{\partial }^{2}}{{\partial x}^{2}}+\frac{1}{{a}_{1}^{2}}\frac{{\partial }^{2}}{{\partial y}^{2}}\right)\theta \left({T}_{1}-{T}_{^\circ }\right)+{Q}_{^\circ }+\frac{Rk}{{a}_{1}^{2}}\left({T}_{1}-{T}_{^\circ }\right)\frac{{\partial }^{2}\theta }{{\partial y}^{2}},$$

$${\tau }_{xx}=\frac{2\delta }{1+{\lambda }_{1}}\left[1+\frac{{\lambda }_{2}c\delta }{{a}_{1}}\left(\left(u+1\right)\frac{\partial }{\partial x}+v\frac{\partial }{\partial y}\right)\right]\frac{\partial u}{\partial x},$$

$${\tau }_{xy}=\frac{1}{1+{\lambda }_{1}}\left[1+\frac{{\lambda }_{2}c\delta }{{a}_{1}}\left(\left(u+1\right)\frac{\partial }{\partial x}+v\frac{\partial }{\partial y}\right)\right]\left(\frac{\partial u}{\partial y}+{\delta }^{2}\frac{\partial v}{\partial x}\right),$$

14 $${\tau }_{yy}=\frac{2\delta }{1+{\lambda }_{1}}\left[1+\frac{{\lambda }_{2}c\delta }{{a}_{1}}\left(\left(u+1\right)\frac{\partial }{\partial x}+v\frac{\partial }{\partial y}\right)\right]\frac{\partial v}{\partial y},$$

$${{\mathrm{\hbar}}}_{1}=1+a{\text{cos}}2\pi x, {{\mathrm{\hbar}}}_{2}=-d-b{\text{cos}}\left(2\pi x+\varnothing \right).$$

From (11), (12) and (13) and by ignoring terms containing $\updelta$ and its higher powers by utilizing the long wavelength approximation ($\updelta$ << 1) and low Reynolds number assumption, we have

15 $$\frac{\partial p}{\partial x}=\frac{1}{\epsilon }\frac{\partial }{\partial y}\left[\frac{1}{1+{\lambda }_{1}}\frac{\partial u}{\partial y}\right]-\frac{1}{Da}\left(u+1\right)+Gr\theta -{M}^{2}\left(u+1\right),$$

and

16 $$\frac{\partial p}{\partial y}=0,$$

17 $$\frac{{d}^{2}\theta }{d{y}^{2}}+{N}_{1}=0.$$

According to Eq. (16), $p$ is not dependent on $y$. Consequently, (15) may be expressed as

18 $$\frac{dp}{dx}=\frac{1}{\epsilon }\frac{\partial }{\partial y}\left[\frac{1}{1+{\lambda }_{1}}\frac{\partial u}{\partial y}\right]-\left(\frac{1}{Da}+{M}^{2}\right)\left(u+1\right)+Gr\theta ,$$

These non-dimensional boundary conditions are^40,41^ equivalent to

$$u=-1, \,at \,y={{\mathrm{\hbar}}}_{1}, {{\mathrm{\hbar}}}_{2},$$

19 $$\theta =1, at\, y={{\mathrm{\hbar}}}_{1}, {{\mathrm{\hbar}}}_{2}.$$

According to the wave frame, the volumetric flow rate in the non-dimensional form is

20 $$F=\int \limits_{{{\mathrm{\hbar}}}_{2}}^{{{\mathrm{\hbar}}}_{1}}udy.$$

## Solution of the problem

We get $\theta$ and $u$ by solving (15), (17), and applying the boundary conditions (19).

21 $$\theta = \frac{{2{\mathrm{\hbar}} _{1} - 2y - {\mathrm{\hbar}} _{1}^{{2_{2} }} N_{1} + {\mathrm{\hbar}} _{1} {\mathrm{\hbar}} _{2}^{2} N_{1} + {\mathrm{\hbar}} _{1}^{2} yN_{1} }}{{2\left( {{\mathrm{\hbar}} _{1} - {\mathrm{\hbar}} _{2} } \right)}},$$

22 $$u={b}_{^\circ }{e}^{-\sqrt{{A}_{2}}y}\left\{{b}_{1}+{b}_{2}{e}^{2\sqrt{{A}_{2}}y}+{b}_{4}\left({b}_{3}-{e}^{\sqrt{{A}_{2}}\left(2{{\mathrm{\hbar}}}_{1}+y\right)}+{e}^{\sqrt{{A}_{2}}\left(2{{\mathrm{\hbar}}}_{2}+y\right)}+{e}^{\sqrt{{A}_{2}}\left({{\mathrm{\hbar}}}_{1}+2y\right)}-{e}^{\sqrt{{A}_{2}}\left({{\mathrm{\hbar}}}_{2}+2y\right)}\right)+{b}_{5}+{b}_{6}{e}^{\sqrt{{A}_{2}}\left({\mathrm{\hbar}}+2y\right)}+{b}_{7}\left({{\mathrm{\hbar}}}_{1}-y\right)\left({b}_{8}+{b}_{9}y\right)\left({e}^{\sqrt{{A}_{2}}\left({2{\mathrm{\hbar}}}_{2}+y\right)}-{e}^{\sqrt{{A}_{2}}\left({2{\mathrm{\hbar}}}_{1}+y\right)}\right)\right\} ,$$

where $${N}_{1}=\frac{\beta }{1+R}, {b}_{^\circ }=\frac{1}{2{A}_{2}^{2}{(e}^{2\sqrt{{A}_{2}}{\mathrm{\hbar}}_{1}}-{e}^{2\sqrt{{A}_{2}}{\mathrm{\hbar}}_{2}})},{b}_{1}=-2{A}_{2}^{2}{e}^{2\sqrt{{A}_{2}}{{({\mathrm{\hbar}}}_{1}+{\mathrm{\hbar}}}_{2})}\left({e}^{\sqrt{{A}_{2}}{\mathrm{\hbar}}_{1}}-{e}^{\sqrt{{A}_{2}}{\mathrm{\hbar}}_{2}}\right),$$ $${b}_{2}=-2{A}_{2}^{2}\left({(e}^{\sqrt{{A}_{2}}{\mathrm{\hbar}}_{1}}-{e}^{\sqrt{{A}_{2}}{\mathrm{\hbar}}_{2}}\right),{b}_{3}=\left({e}^{\sqrt{{A}_{2}}{{(2{\mathrm{\hbar}}}_{1}+{\mathrm{\hbar}}}_{2})}-{e}^{\sqrt{{A}_{2}}{{({\mathrm{\hbar}}}_{1}+2{\mathrm{\hbar}}}_{2}}\right),$$

$${b}_{4}=2{A}_{3}{({{\mathrm{\hbar}}}_{1}-{{\mathrm{\hbar}}}_{2})}^{2}{N}_{1}+2{A}_{1}{A}_{2}, {b}_{5}=-2{A}_{2}{A}_{3}{({{\mathrm{\hbar}}}_{1}-{{\mathrm{\hbar}}}_{2})}^{2}{e}^{\sqrt{{A}_{2}}{{(2{\mathrm{\hbar}}}_{1}+{\mathrm{\hbar}}}_{2})},$$

$${b}_{6}=2{A}_{2}{A}_{3}{({{\mathrm{\hbar}}}_{1}-{{\mathrm{\hbar}}}_{2})}^{2},{b}_{7}=-{A}_{2}{A}_{3}({{\mathrm{\hbar}}}_{1}-{{\mathrm{\hbar}}}_{2}),{b}_{8}=2-{{\mathrm{\hbar}}}_{1}{{\mathrm{\hbar}}}_{2}{N}_{1}+{h}_{2}^{2}{N}_{1}, {b}_{9}=\left({{\mathrm{\hbar}}}_{1}-{{\mathrm{\hbar}}}_{2}\right){N}_{1},$$

$$m=\epsilon \left(1+{\lambda }_{1}\right),{A}_{1}=m\left(\frac{\partial p}{\partial x}+\frac{1}{Da}+{M}^{2}\right),{A}_{2}=m\left(\frac{1}{Da}+{M}^{2}\right), {A}_{3}=mGr.$$

It follows from (22) and (20) that the pressure gradient can be written as

23 $$\frac{dp}{dx}=\frac{1}{{v}_{2}}\left[F+\sqrt{{A}_{2}}{b}_{^\circ }\left({b}_{1}-{b}_{2}+{b}_{5}\right){e}^{-\sqrt{{A}_{2}(}{{\mathrm{\hbar}}}_{1}-{{\mathrm{\hbar}}}_{2})}-\sqrt{{A}_{2}}{b}_{^\circ }{b}_{6}{e}^{\sqrt{{A}_{2}}{{\mathrm{\hbar}}}_{1}}+{b}_{10}{{\mathrm{\hbar}}}_{2}\left({b}_{8}+{b}_{9}\left({{\mathrm{\hbar}}}_{1}-{{\mathrm{\hbar}}}_{2}\right)\right)\left(1-{b}_{9}\right)\right]-\left(\frac{1}{Da}+{M}^{2}\right),$$

where $${b}_{10}={b}_{7}\left({e}^{2\sqrt{{A}_{2}}{\mathrm{\hbar}}_{2}}-{e}^{2\sqrt{{A}_{2}}{\mathrm{\hbar}}_{1}}\right), {v}_{1}=\sqrt{{A}_{2}}{b}_{^\circ }{b}_{3}{e}^{-\sqrt{{A}_{2}}{({\mathrm{\hbar}}}_{1}-{\mathrm{\hbar}}_{2})}+\sqrt{{A}_{2}}{b}_{^\circ }\left[{e}^{\sqrt{{A}_{2}}{(2{\mathrm{\hbar}}}_{1}-{\mathrm{\hbar}}_{2})}-{e}^{\sqrt{{A}_{2}}{\mathrm{\hbar}}_{1}}\right]-{b}_{^\circ }{({\mathrm{\hbar}}}_{1}-{\mathrm{\hbar}}_{2}){e}^{2\sqrt{{A}_{2}}{\mathrm{\hbar}}_{1}}+{b}_{^\circ }{({\mathrm{\hbar}}}_{1}-{\mathrm{\hbar}}_{2}){e}^{2\sqrt{{A}_{2}}{\mathrm{\hbar}}_{2}},{v}_{2}=2m{A}_{2}{v}_{1}.$$

Based on (23) we have the pressure rise $\Delta {p}_{\lambda }$ per one wavelength and the friction force for the upper and lower wall ${F}_{\lambda }^{u},{F}_{\lambda }^{L}$ as follow:

$$\Delta {p}_{\lambda }={\int }_{0}^{1}\left(\frac{dp}{dx}\right)dx,{\mathrm{F}}_{\uplambda }^{\mathrm{u}}={\int }_{0}^{1}{{\mathrm{\hbar}}}_{1}^{2}\left(\frac{-dp}{dx}\right)dx\text{ and }{\mathrm{F}}_{\uplambda }^{\mathrm{L}}={\int }_{0}^{1}{{\mathrm{\hbar}}}_{2}^{2}\left(\frac{-dp}{dx}\right)dx.$$

The relation between the velocity components (*u*, *v*) and the stream function $\Psi$ is given by

24 $$u=\frac{\partial\Psi }{\partial y},v=-\frac{\partial\Psi }{\partial x}.$$

From (22) and (24), one can write

$$\Psi =\int udy.$$

## Numerical procedure

Using the command DSlove in the Mathematica program, linear Eqs. (17) and (18) were solved with boundary conditions (19). This procedure is useful in reducing CPU per evaluation as well as reducing error. Also, to obtain graphical solutions and numerical calculations, an appropriate algorithm has been developed for this matter.

## Numerical results and discussion

**Figure 2:**
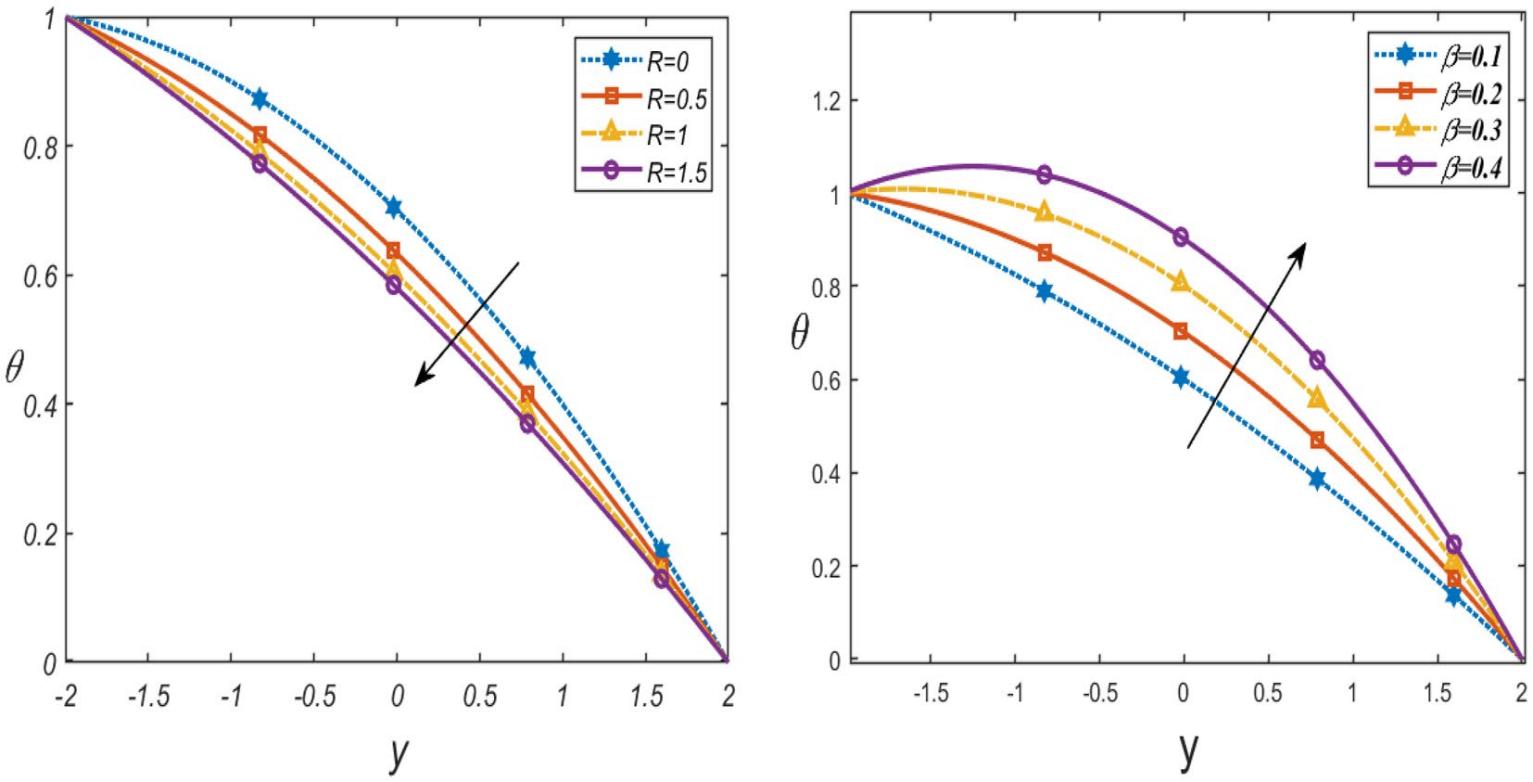
Changes of $\beta , R$ on the temperature $\theta$ against $y$-axis.

This section discusses the graphical data that were found in this study for temperature, velocity, pressure gradient, pressure rise, friction forces, stress and streamlines. MATLAB is used for the simulation and the results are exhibited through graphs. For numerical computations, the material properties of parameter values as in Reddy^40^ are taken under consideration. Figure 2 depict the impact of thermal radiation and heat source/sink on temperature with respect to $y$-axis, while keeping other parameters fixed. As increasing the parameter $\beta$ the temperature increases, while it decreases when the value of the parameter $R$ increasing. The temperature satisfies the boundary conditions. This result is in good agreement with the results obtained by Hayat et al.^6^.

**Figure 3:**
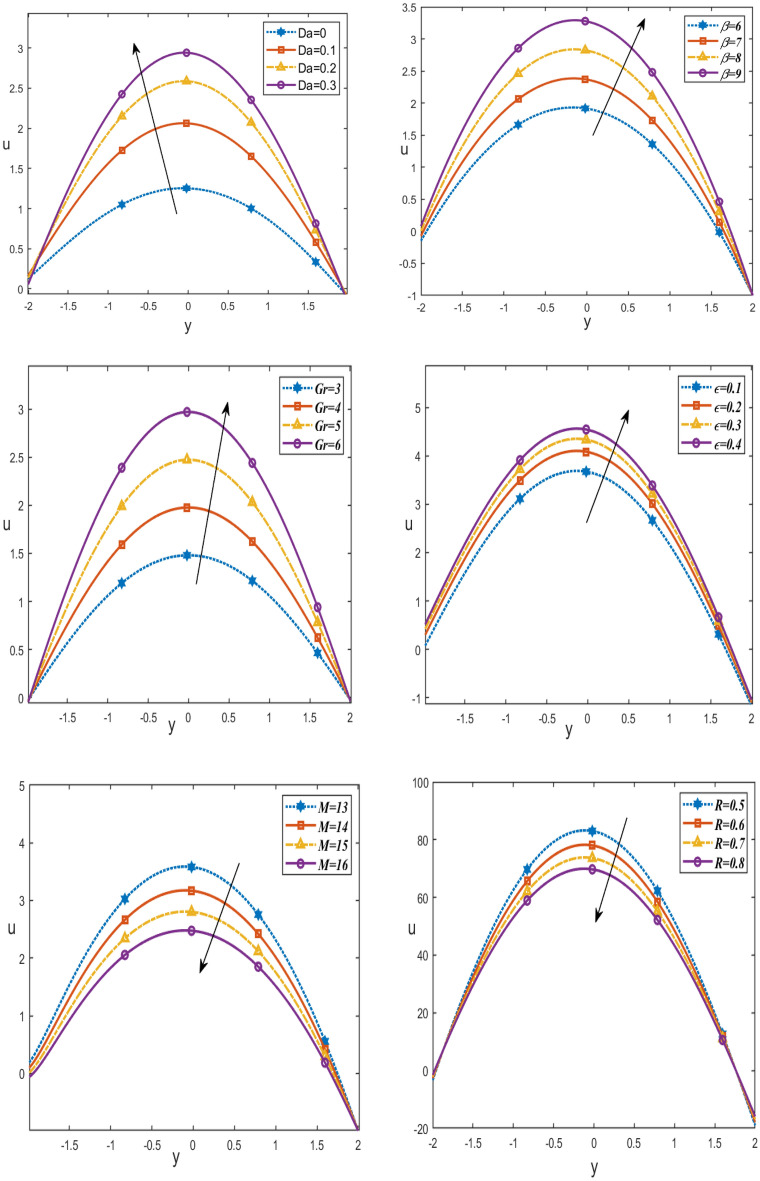
Changes of $Da, \beta , Gr \epsilon, M, R$ on the velocity distribution *u* against $y$-axis.

Figure 3 displays the change in velocity $u$ with the y-axis according to $\epsilon$ is the porosity of the porous medium, thermal radiation $R$, $Gr$ Grashof number, $M$ Hartmann number, heat source/sink $\beta$ and Darcy’s number $Da$. It can be seen that increasing $Da, \beta , Gr$ and $\epsilon$ increases the velocity $u$, while rising $R$ and $M$ cause it to decrease. Moreover, it has the highest value in the channel’s middle and the lowest value at the channel's edges. Also, it satisfies the boundary conditions. This is in good agreement with what was obtained in clinical practice because the nutrients diffuse out of the blood vessels to neighboring tissues^23^.

**Figure 4:**
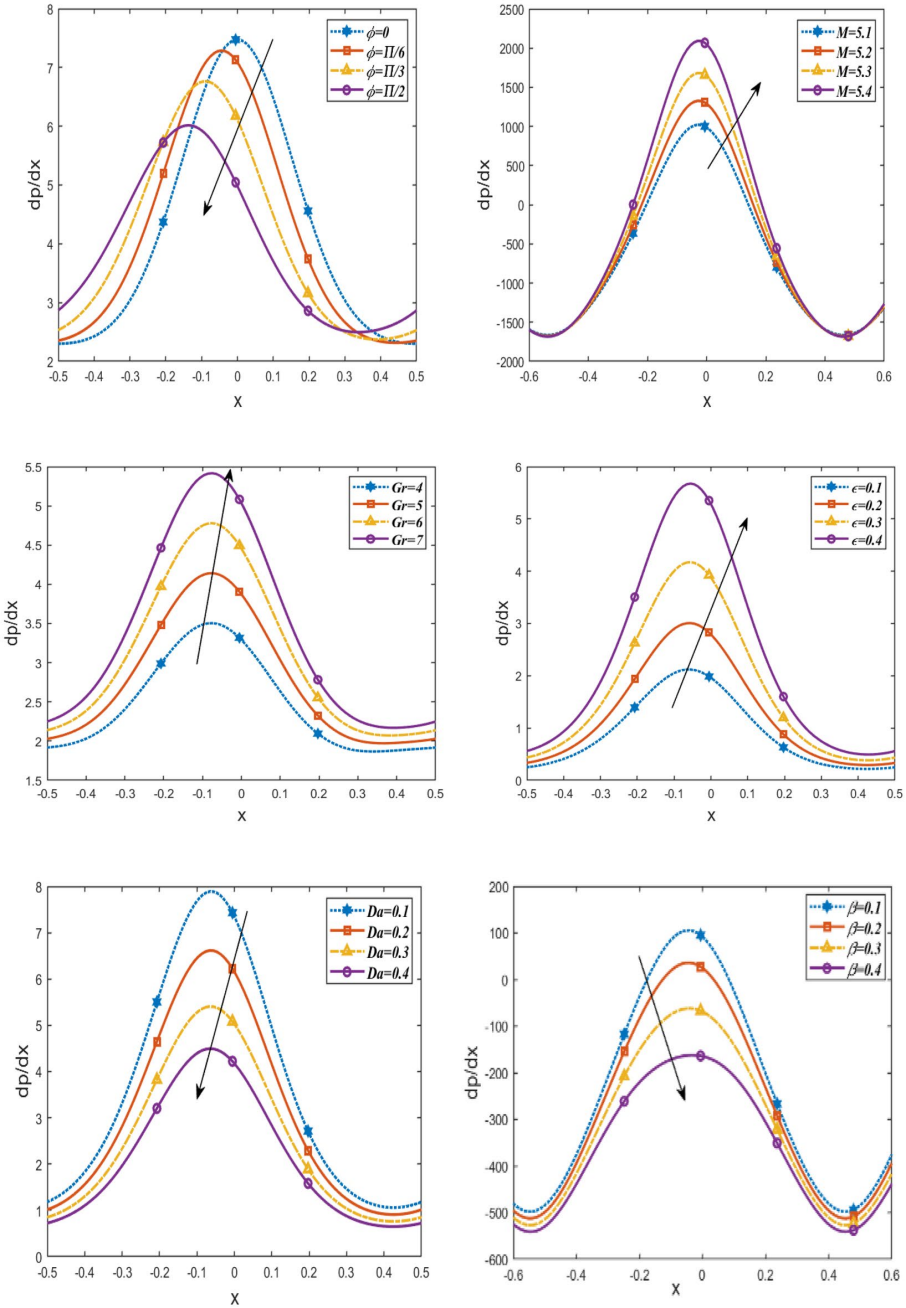
Changes of $Da, \beta , Gr \epsilon ,M, \phi$ on the pressure gradient $\frac{dp}{dx}$ against $x$-axis.

Figure 4 illustrates the variations of the pressure gradient $\frac{dp}{dx}$ with respect to the $x$-axis for various parameters $Da, \beta , Gr,\epsilon , M$ and the phase difference $\phi$. According to the graph, the pressure gradient rises when $M, \epsilon , Gr$ are increased while falling when $Da, \beta , \phi$ are decreased. it is observed that the pressure gradient oscillates in the whole range x. For more authenticity, this result is in good agreement with the results obtained by Reddy^40^.

**Figure 5:**
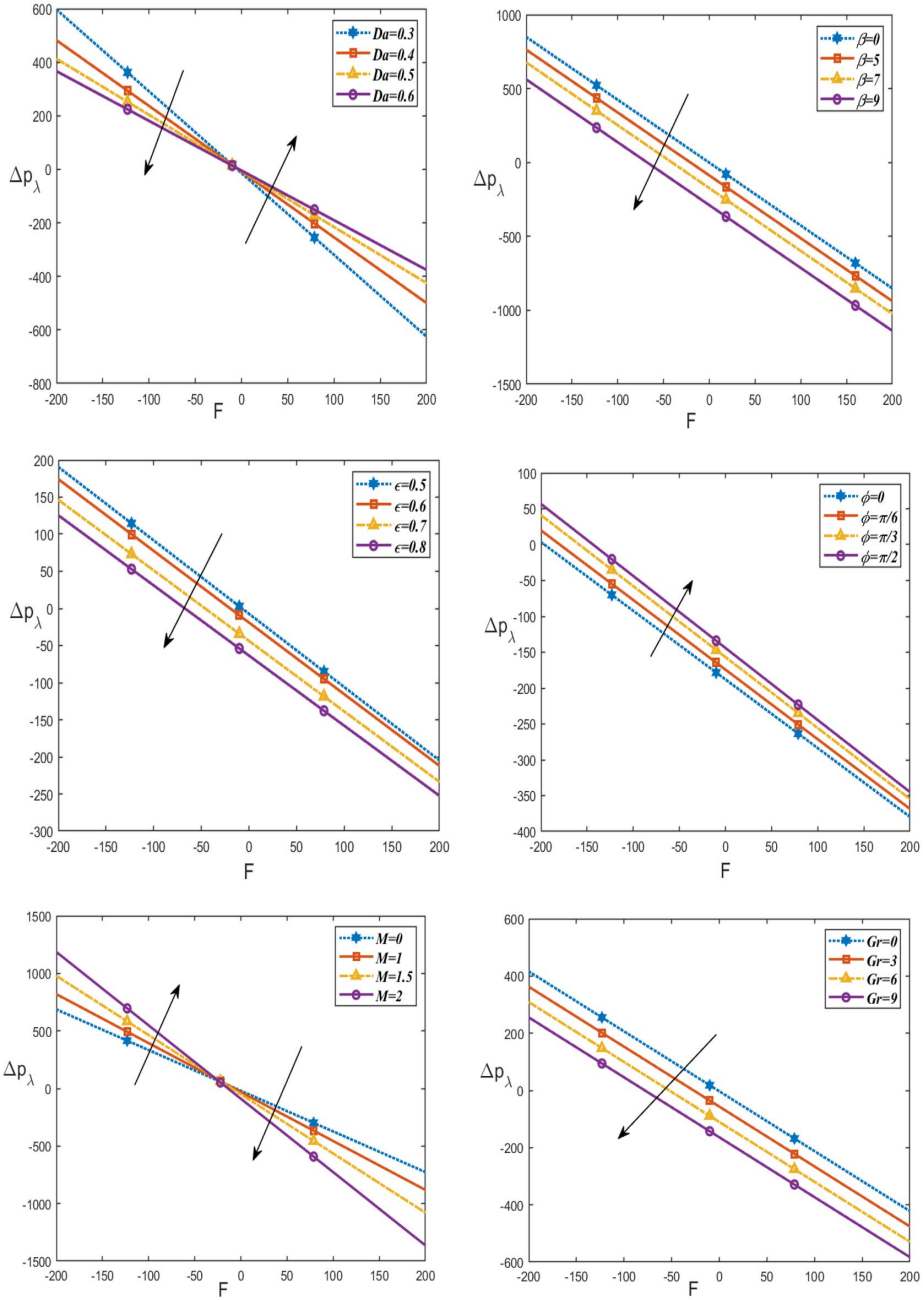
Changes of $Da, \beta , Gr \epsilon ,M, \phi$ on the pressure rise $\Delta {p}_{\lambda }$ against $F$.

Figure 5 presents the impact of $Da, \beta , Gr,\epsilon , M$ and $\phi$ on the pressure rise $\Delta {p}_{\lambda }$ with respect to rate volume flow F. It is noticed that the pressure rise decreases with increasing $\beta , Gr,\epsilon$, while it increases with increasing $\phi$, as well, it increases with increasing $M$ in the region (− 200 ≤ F ≤ 0) and it decreases in the interval (0 ≤ F ≤ 200), otherwise it falls with rising $Da$ in the period (− 200 ≤ F ≤ 0) and increases in the interval (0 ≤ F ≤ 200). This result is in good agreement with the results obtained by Reddy^40^.

**Figure 6:**
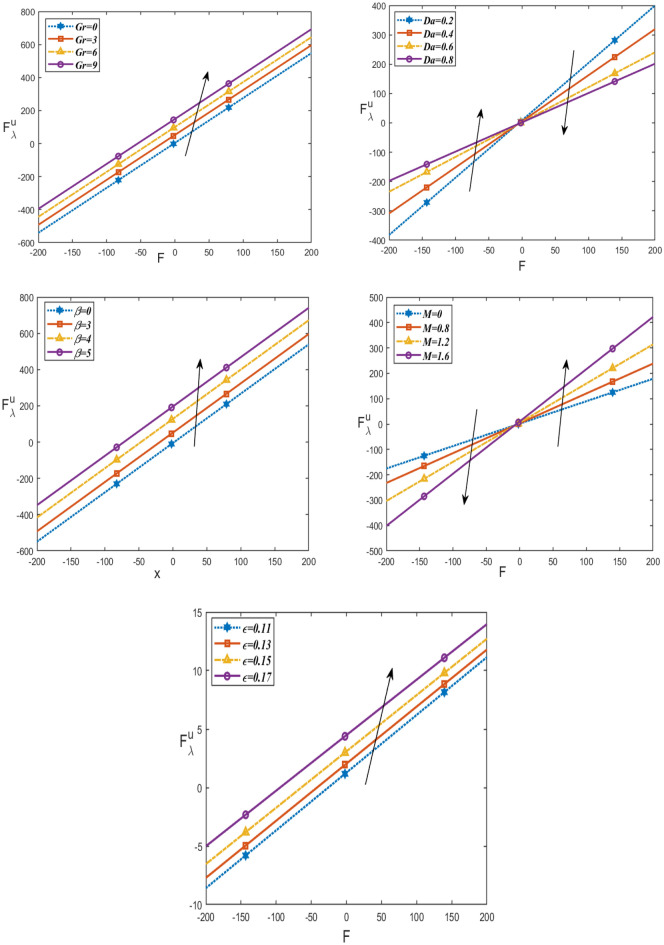
Changes of $Da, \beta , Gr \epsilon ,M$ on the friction force upper ${\mathrm{F}}_{\uplambda }^{\mathrm{u}}$ against *F*.

**Figure 7:**
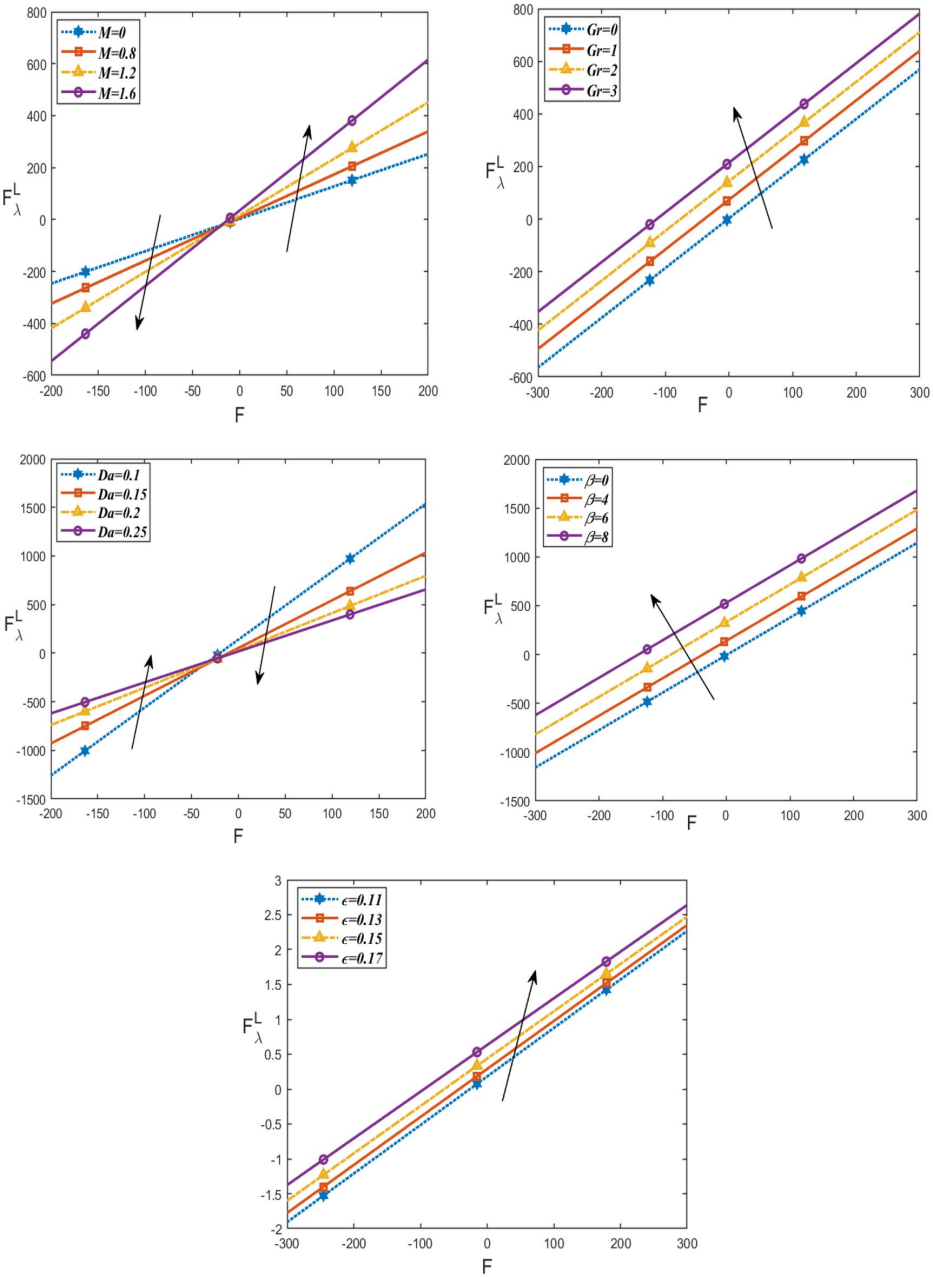
Changes of $Da, \beta , Gr \epsilon ,M$ on the friction force lower ${\mathrm{F}}_{\uplambda }^{\mathrm{L}}$ against *F*.

**Figure 8:**
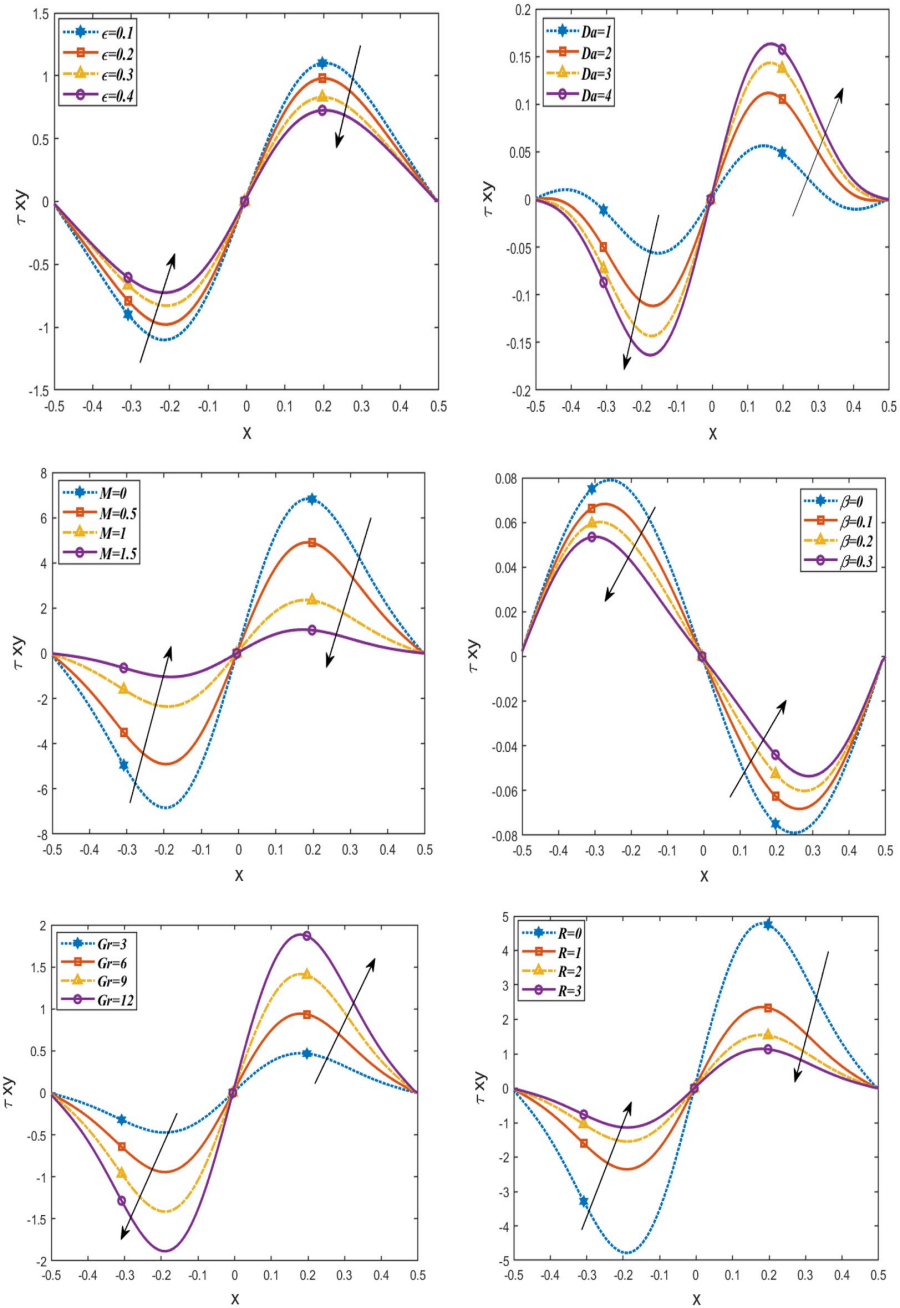
Changes of $Da, \beta , Gr \epsilon ,M, \phi$ on the stress ${\tau }_{xy}$ against $x$-axis.

**Figure 9:**
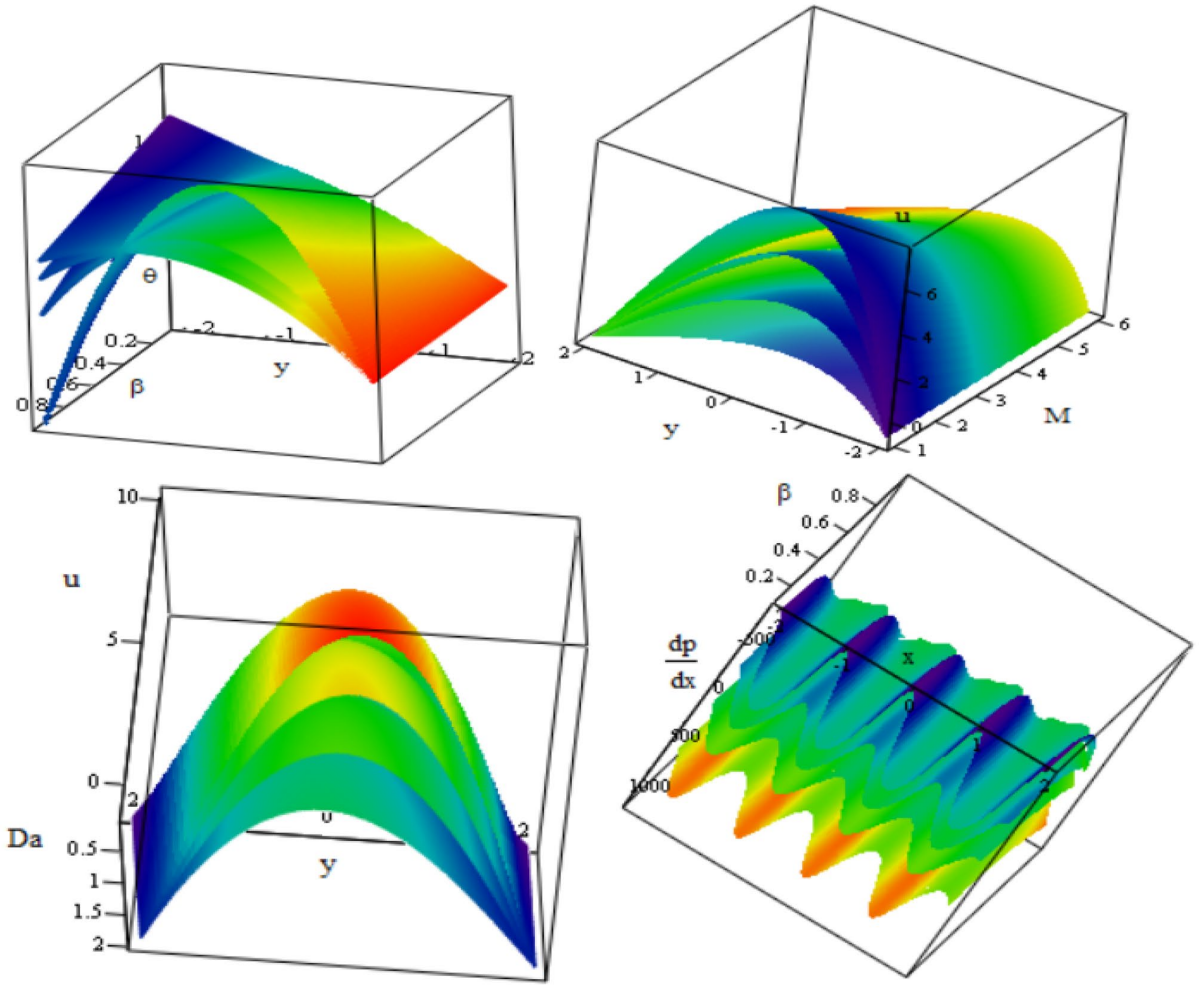
3D plot of temperature *θ,* velocity *u* and the pressure gradient $\frac{dp}{dx}$ with x, y axes for changes in *β*, *M* and *Da* respectively.

Figures 6 and 7 demonstrate how the friction force for upper ${\mathrm{F}}_{\uplambda }^{\mathrm{u}}$ and lower ${\mathrm{F}}_{\uplambda }^{\mathrm{L}}$ varies with respect to a flow rate in volume F under these parameters $Da, \beta , Gr,\epsilon , M$. It is noticed that the friction force for lower and upper increases with increasing $\beta , Gr,\epsilon$, as well, they increase with increasing $Da$ in the interval (− 200 ≤ F ≤ 0) and decrease in the interval (0 ≤ F ≤ 200). But by increasing $M$, they decrease in the interval (− 200 ≤ F ≤ 0) and increase in the interval (0 ≤ F ≤ 200). The behavior of pressure rise is observed to be the inverse of the behavior of friction forces in the upper and lower layers. Figure 8 shows the variations of the shear stress ${\tau }_{xy}$ with respect to x-axis for different values of $\epsilon , Gr, M, \beta , R$ and $Da$. The stress decreases in the interval ($- 0.5 \le x \le 0$) and increases in the interval ($0 \le x \le 0.5$) by rising $Gr, Da,$ and $\beta$. While, it increases in the interval ($- 0.5 \le x \le 0$) and decreases in the interval ($0 \le x \le 0.5$) by rising g $\epsilon , R, M$. Figure 9 shows the 3D schematics concern θ, u, and $\frac{dp}{dx}$, with regards to $x$ and $y$ axes under the effect of heat source $\beta$, Hartman number $M$, and Darcy number $Da$. The temperature is observed to drop as $R$ increases, while the velocity increases with increasing $Da$ and decreases with increasing $M$. Also, the pressure gradient decreases with increasing $\beta$. In 3D, all physical quantities derived from peristaltic flow overlap and dampen as they increase in order for particles to reach equilibrium. Most physical fields move in peristaltic flow, which is more relevant for the vertical distance of the curves that were created.

## Streamlines pattern and trapping phenomenon

**Figure 10:**
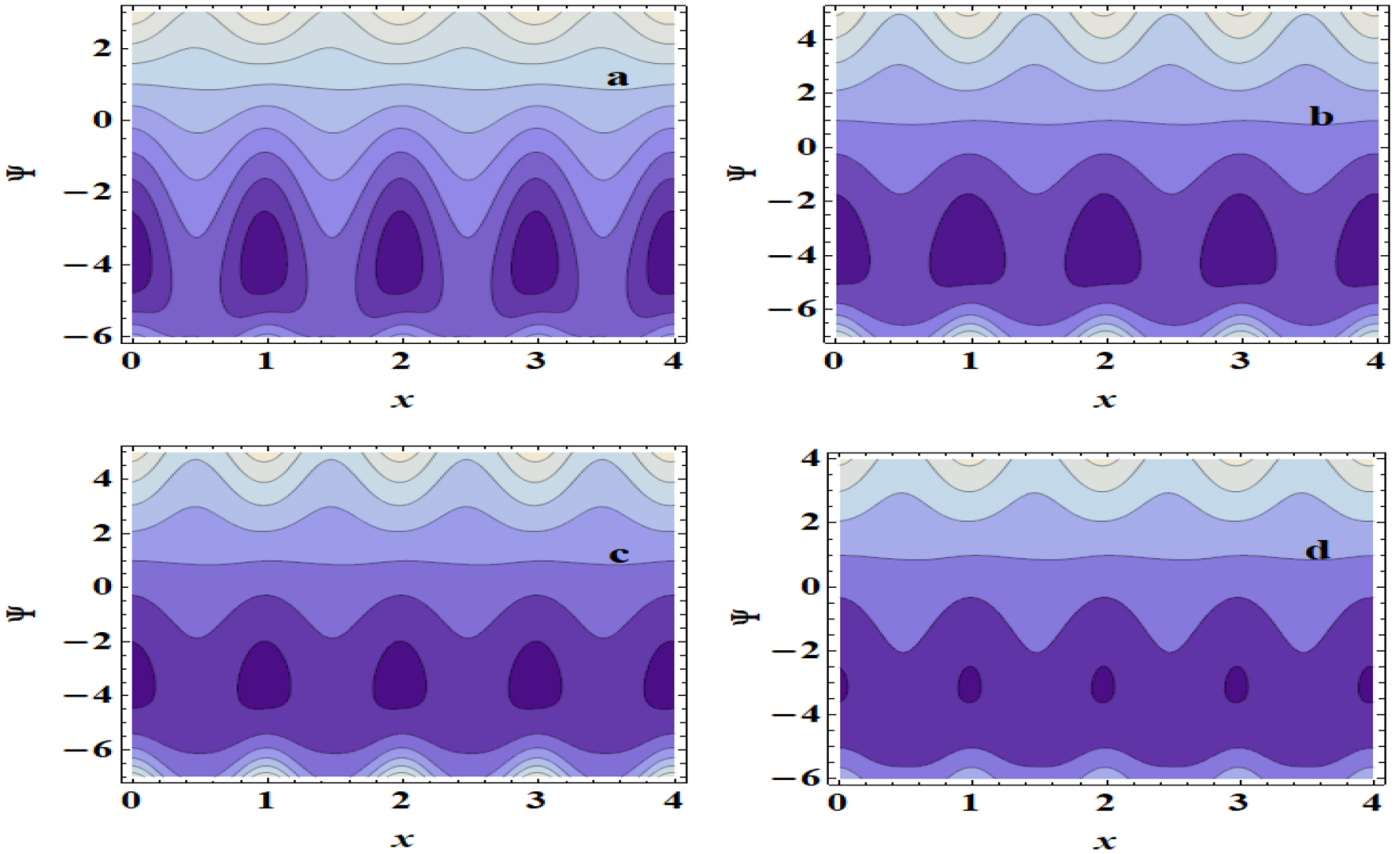
Streamlines for (**a**) $$\beta$$ = 0, (**b**) $$\beta$$ = 0.1, (**c**) $$\beta$$ = 0.3, (**d**) $$\beta$$ = 0.5.

**Figure 11:**
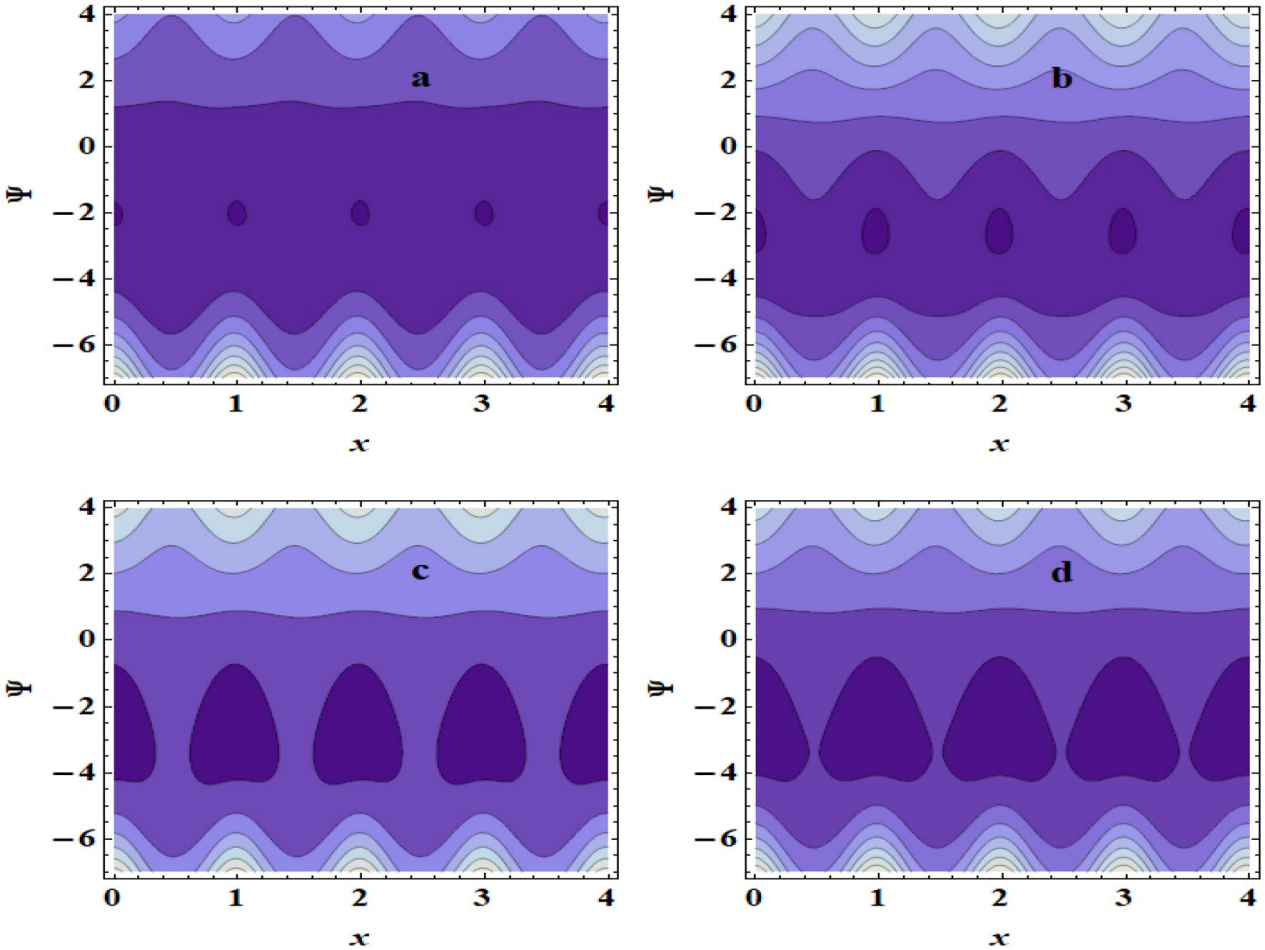
Streamlines for (**a**) $$Gr$$ = 1, (**b**) $$Gr$$ = 3, (**c**) $$Gr$$ = 5, (**d**) $$Gr$$ = 7.

**Figure 12:**
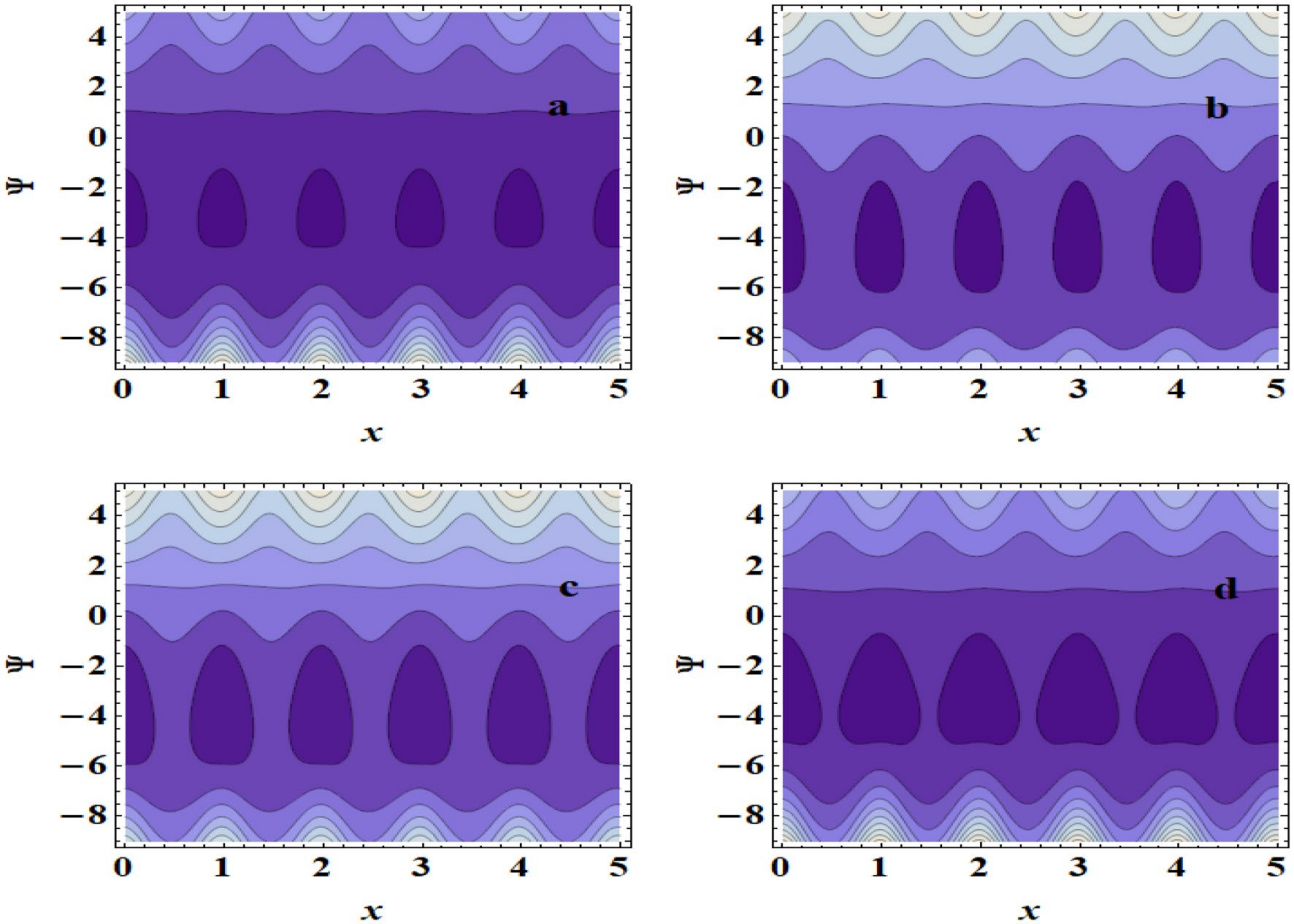
Streamlines for (**a**) $$M$$ = 0, (**b**) $$M$$ = 0.1, (**c**) $$M$$ = 0.5, (**d**) $$M$$ = 0.8.

The peristaltic mechanism includes a study of the trapping phenomenon. Few streamlines shut during peristalsis, causing the creation of a bolus that circulates inside and advances at the rate of the peristaltic waves. This occurrence is known as trapping. Now, we will discuss this interesting case under the influence of some influences such as the heat source/sink $\beta$, the Grashof number $Gr$ and the Hartman number $M$. Surprisingly, we observe that the trapping phenomenon occurs as shown in Fig. 10a–d, where it is when the value of the heat source/sink $\beta$ increases that the bolus size decreases. In addition to, In Fig. 11a–d, it is seen that the boluses increase in size with increasing $Gr$. Also, we found that as $M$ is raised, the trapped bolus’s size grows, as shown in Fig. 12a–d. This increase in the size describes the volume of the fluid that is bounded by invariant closed streamlines. Furthermore, compared to the symmetric channel, the size of the trapped bolus is less in the asymmetric channel.

## Conclusion and future work

The role of the magnetic field and heat transfer have been studied for a fluid that is not Newtonian in a porous medium. To solve the problem mathematically, small Reynolds number and long wavelength assumptions are utilized. Graphical illustrations have been used to explain and discuss the impact of different physical factors on the flow characteristics. Below is a list of the important results.

- The partial differential equations that appear in this paper have an accurate analytical solution approach.
- Temperature can be increased by increasing $\beta$ and reduced by increasing $R$.
- The flow has a maximum velocity in the centre and subsequently increases with increasing β, Da, Gr, $\epsilon$ and drops with increasing $M, R$, according to the graphical solutions for the velocity.
- The magnitude of pressure gradient has an oscillating behaviour as it increases by increments of $M, Gr, \epsilon$ and decreases by increments of $Da , \phi$.
- Parameters $Gr, \epsilon$, $\beta$ have a decreasing effect on the pressure rise, while parameter $\phi$ has an increasing effect on it, as well, it increases with increasing $M$ in the interval − 200 ≤ F ≤ 0 and decreases in the interval 0 ≤ F ≤ 200, otherwise it drops with raising $Da$ in the period − 200 ≤ F ≤ 0 and increases in the interval 0 ≤ F ≤ 200.
- When compared to the pressure rise, the frictional force similarly has the opposite trend.
- The volume of the trapped bolus increases as the magnetic field and Grashof number increase, while it decreases as the heat source increases.
- Researchers working in the domains of science, engineering, medicine, and fluid mechanics may find the study’s findings beneficial.
- Future research may be done in this approach to examine how slip circumstances affect flow characteristics.

## Data Availability

The datasets used and/or analyzed during the current study available from the corresponding author on reasonable request.

## List of symbols

$\alpha_{1} ,{\upgamma }_{1}$: Amplitudes of the wavy walls
$${\text{a}},{\text{b}}$$: Amplitude ratios
$\alpha_{1} + \alpha_{2}$: Width of channel
$$\lambda$$: Wavelength
c: Wave speed
t*′*: Time
u: Axial velocity
$B_{^\circ }$: Intensity of external magnetic field
$$\rho$$: Constant density
g: Acceleration due to gravity
K: Thermal conductivity
$$c_{p}$$: Specific heat
M: Hartmann number
P: Pressure
u*′*, v*′*: Velocity components in wave frame
$$\mu_{^\circ }$$: Constant viscosity
R: Thermal radiation parameter
$\sigma$: Electrical conductivity of the fluid
Re: Reynolds number
Gr: Grashof number
$\beta$: Heat source/sink parameter
$$\theta$$: Temperature distribution
$$Da$$: Darcy’s number
$$Q_{^\circ }$$: Heat source
